# Herbivore-Induced Specificity and Diversity in *Piper arboreum* Volatiles

**DOI:** 10.3390/plants15020290

**Published:** 2026-01-18

**Authors:** Mariana A. Stanton, Variluska Fragoso, Lydia F. Yamaguchi, Massuo J. Kato

**Affiliations:** 1 Laboratory of Natural Products Chemistry, Institute of Chemistry, University of São Paulo, Av. Prof. Lineu Prestes, 748, Butantã, São Paulo 05508-000, SP, Brazil; 2 Instituto de Pesquisas Ambientais (IPA), Secretaria de Meio Ambiente, Av. Miguel Estefno, 3687, Água Funda, São Paulo 04301-902, SP, Brazil; 3 Instituto de Pesquisas Tecnológicas do Estado de São Paulo, Av. Prof. Almeida Prado, 532, Butantã, São Paulo 05508-901, SP, Brazil

**Keywords:** volatile organic compounds, herbivore-induced plant volatiles, *Piper*, herbivores, generalist, specialist

## Abstract

The essential oils obtained by steam distillation of leaves of *Piper* species have found several applications in bioeconomy due to their various biological properties. Nevertheless, the analysis of essential oils does not provide information regarding the ecologically relevant volatile organic compounds (VOCs) emitted by metabolically active leaves under real-world conditions, challenged or not by herbivore damage. In this study, *P. arboreum* growing in a highly diverse area was observed as the host of two generalist caterpillars—*Gonodonta maria* (Erebidae) and *Dysodia spissicornis* (Thyrididae)—and one *Piper*-specialist from the genus *Eois* (Geometridae). The effect of the leaf attack caused by the three different caterpillars on VOCs emission indicated significant and herbivore-specific changes in leaf-induced responses. The profiles of undamaged leaves showed that the two generalist herbivores induced a higher number of single VOCs and of total VOCs emissions by *P. arboreum* when compared to the herbivory of the specialist caterpillar. Many of the VOCs emitted by herbivore-damaged leaves contained terpenoids that have been previously shown to attract parasitoids, such as (*E*)-β-ocimene, linalool, DMNT and (*E*)-β-caryophyllene. All three herbivores significantly altered the VOC profile of *P. arboreum* leaves compared to undamaged controls, but specific composition signatures were observed, highlighting the complexity of chemical communication at multitrophic levels.

## 1. Introduction

Approximately 1000 species of *Piper* are found worldwide. Of these, around 500 species are native to Brazil, where they grow predominantly in tropical rainforests and riparian forests and can account for up to 15% of the species in the understory [1]. Increased (non-volatile) secondary metabolite diversity in *Piper* sp. has been related to an increased specialization of associated herbivores and increased diversity of their parasitoid enemies in natural ecosystems [2]. In addition, decreased volatile diversity in *Piper* sp. communities has been correlated to higher herbivory in a field study [3]. However, this report did not control the timing of herbivore damage on the leaves analyzed, which may account for the lack of volatile variation, as herbivore-induced plant volatiles can be rapid and transient responses to herbivory [4]. Another field study found evidence that an increase in *Piper* volatile diversity decreased specialist herbivory [5], although these authors measured volatiles by extracting leaves with solvent. In parallel, there is an extensive literature on essential oil characterization of *Piper* volatiles (e.g., [6,7]). However, essential oils do not always accurately represent the volatile blends that are actually released into the air by the plant, and their composition is related to harsh distillation conditions and residual leaf enzymatic activity [8]. Nonetheless, a previous study showed that essential oils of *Piper mollicomum* attracted predatory wasps in the field, and some of the compounds were also present in volatile blends emitted from artificially damaged leaves in the field [9].

To our knowledge, no studies have attempted to collect the volatile headspace of *Piper* plants in the field with natural herbivory, which would provide a more ecologically realistic picture of what compounds are encountered by foraging herbivores and their natural enemies, such as predators and parasitoids [10,11]. Furthermore, most studies of herbivore-induced VOCs (volatile organic compounds) have focused on crop plants given the interest in biological control and agricultural push-pull strategies, and few studies have been carried out with native plants and in high biodiversity tropical habitats [11,12].

In the present study, we collected the volatile headspace [13] of *Piper arboreum* plants in the field to evaluate if there are changes in its volatile profile after herbivory by three Lepidopteran herbivores. Such herbivores are found feeding on *P. arboreum* in nature and differ in diet breadth and peak seasonal abundance. *P. arboreum* Aubl. (Piperaceae) is a neotropical perennial shrub or small tree found in populations in the understory of tropical forests and riparian vegetation throughout Brazil, with a distribution ranging from Central to South America. The study was carried out in a protected area, which is situated in a transition zone between the Cerrado savannah and Atlantic Forest biomes in southeastern Brazil, at the Mogi-Guaçu Biological Reserve (hereafter REBIO Mogi-Guaçu) in the state of São Paulo. Previous analysis of *P. arboreum* volatile constituents by essential oil extraction showed that its main volatile compounds are monoterpenes and sesquiterpenes, but there is significant compositional variation between samples collected at different sites and between studies [14,15]. There is also evidence of parasitism on herbivores of this plant [16,17], but little is known regarding the emission of VOCs by *P. arboreum* under natural field conditions in which these ecological interactions occur. Additionally, there is no evidence whether there is variation in the emission of VOCs depending on the herbivore identity, which could potentially affect parasitoid attraction.

Previous preliminary surveys of immature Lepidopteran herbivores on *P. arboreum* suggested that they differ in their preference for feeding on young vs. mature leaves, and in the timing of their peak seasonal abundance in the field. Larvae of the generalist moth *Gonodonta maria* (Lepidoptera: Erebidae) typically feed on developing (young) leaves, and caterpillars of *Dysodia spissicornis* (Lepidoptera: Thyrididae) are generalist leaf-rollers that usually feed on fully developed (mature) leaves of *P. arboreum*. Both generalist moths are found more frequently at one particular field site in Brazil (Mogi-Guaçu Biological Reserve, or REBIO Mogi-Guaçu) in the warm rainy season (November–March). In contrast, the *Piper*-specialist larvae from the genus *Eois* (Lepidoptera: Geometridae) typically feed on mature leaves of *P. arboreum* at the beginning of the cold dry season (April–July). This higher occurrence of specialized herbivores feeding on *Piper* species, including *P. arboreum*, during the transition between the rainy and dry seasons has been observed for *Eois* sp. in other seasonal tropical forests in Brazil [17,18,19]. Preliminary species delimitation of the *Eois* specimens collected on *P. arboreum* at this field site places them in what would be considered part of the Olivaceae clade (as defined in [20]). However, recent studies on this genus support the existence of cryptic species within this clade [21]. The samples from the present study belong to a single species included in what was called the “Hyperythraria clade” [21,22], based on novel molecular data (Molecular Operational Taxonomic Unit or MOTU, defined by DNA barcoding) and on the dark green wing pattern shared by *Eois hyperytharia* and *E. goodmanii*. Although the wing color pattern suggests that these specimens are genetically closer to the *E. goodmanii* MOTU, they are likely a distinct novel species. Further genetic sequencing and morphological studies are necessary to confirm its taxonomic identity, which is beyond the scope of the present study, and the novel species shall hereafter be referred to as *Eois* “near” *goodmanii* (*E.* nr *goodmanii*).

The specific goals of this study were to address the following questions in nature: (1) Do three native moth herbivores that differ in diet breadth elicit specific VOC profiles in *Piper arboreum*? If so, (2) are herbivore-induced changes in VOC profiles due to quantitative differences (total compounds or sum of compounds emitted), qualitative differences (identity of compounds in profile) or changes in chemical compound diversity (complexity of VOC profile)? And (3) are there changes in leaf VOC pools due to leaf age or season?

## 2. Results

### 2.1. Specificity of Herbivore-Induced Volatiles from Piper arboreum in Nature

**Figure 1:**
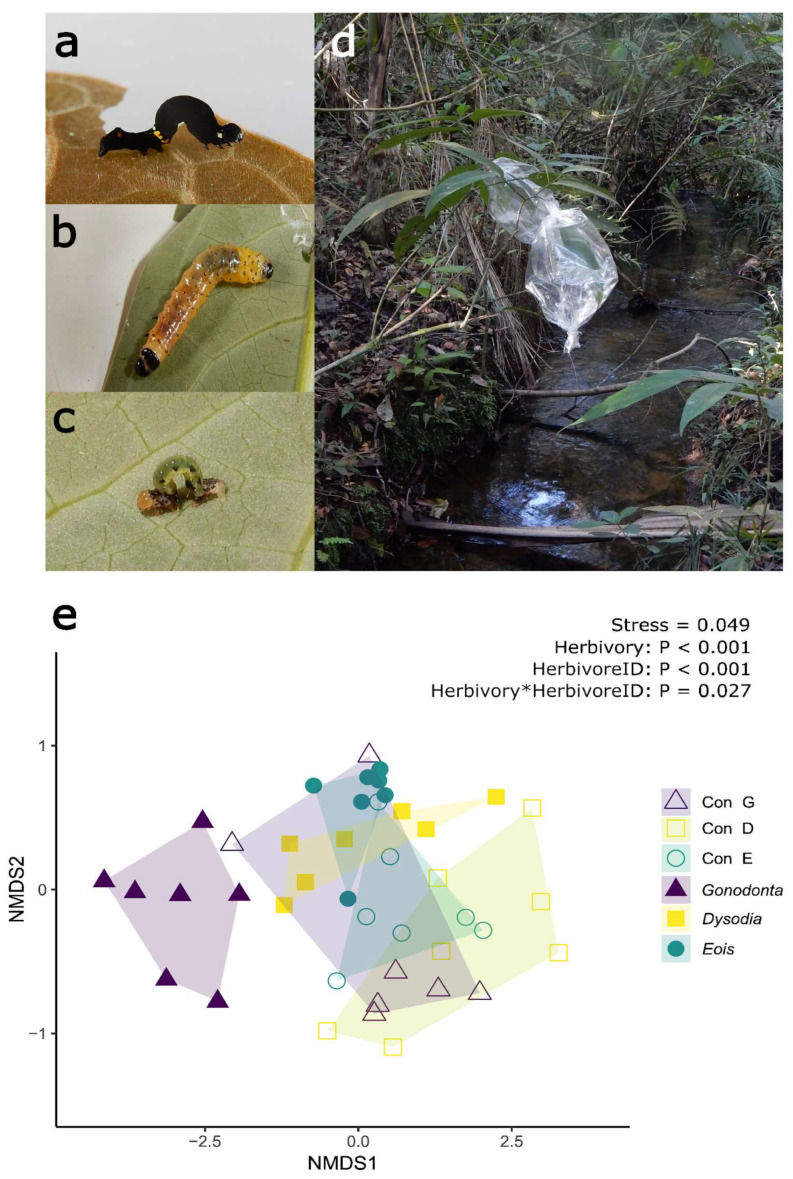
*Piper arboreum* leaves with herbivory by larvae of three native lepidopteran herbivores in the field showing distinct volatile profiles. Larvae of (**a**) *Gonodonta maria* (Erebidae) feed on young (reddish) leaves of *P. arboreum*, while (**b**) *Dysodia spissicornis* (Thyrididae) and (**c**) *Eois* nr *goodmanii* (Geometridae) feed on mature leaves. (**d**) Example of a *P. arboreum* plant with paired volatile collection design using plastic baking foil bags to enclose target leaves and PDMS silicone tubing in the field (see text for details). (**e**) Non-metric multidimensional scaling (NMDS) of volatile blends released by *P. arboreum* plants after attack by three different Lepidopteran herbivores and their respective controls. A total of *n* = 7 plants were used for each herbivore type (HerbivoreID). Each individual plant generated samples of leaves with herbivores and control leaves without herbivores from distal branches (see text for details on VOC sampling). Full symbols represent leaves with herbivory by *Gonodonta maria* (“*Gonodonta*”), *Dysodia spissicornis* (“*Dysodia*”) and *Eois hyperythraria* (“*Eois*”). Open symbols represent respective undamaged control leaves for *G. maria* (“Con G”), *D. spissicornis* (“Con D”) and *E. hyperythraria* (“Con E”). Two-dimensional stress value for NMDS and *p*-values for the PERMANOVA analysis are shown in the upper right corner.

To test whether herbivory by larvae of three different native Lepidoptera species changed the volatile profiles of *P. arboreum* leaves, volatile sampling was carried out using polydimethylsiloxane (PDMS) silicone tubing [23]. This sampling method is well suited for field sampling, as it does not require the use of vacuum pumps, and headspace volatiles can be more readily collected in the field. Additionally, samples can be easily transported back to the laboratory for analysis by gas chromatography and mass spectrometry (GC-MS). Figure 1a–d shows the three types of herbivore larvae used and an example of the field volatile sampling using plastic baking foil bags to enclose target leaves together with the PDMS tubes (see Methods for further details on volatile sampling).

Analysis of leaf volatile profiles by non-metric multidimensional scaling (NMDS) revealed that herbivory by *Gonodonta maria* larvae (hereafter, *Gonodonta* herbivory), *Dysodia spissicornis* (hereafter, *Dysodia* herbivory) and *Eois* nr *goodmanii* (hereafter, *Eois* herbivory) led to the emission of different volatile profiles by *P. arboreum* leaves (NMDS, 2D stress = 0.049, Figure 1e). The volatile profiles of paired undamaged control leaves (hereafter ConG, ConD and ConE, for the respective control samples for *Gonodonta*, *Dysodia* and *Eois*) are shown. This change in volatile profile composition is supported by the results of a Permutational Analysis of Variance (PERMANOVA) analysis, which revealed a significant effect of herbivory (Pseudo-F = 6.3743, *p* < 0.001), of herbivore species (herbivoreID, Pseudo-F = 4.4193, *p* < 0.001) and a significant interaction between herbivory and herbivoreID (Pseudo-F = 2.4174, *p* = 0.027) on *P. arboreum* leaf volatile profiles (Figure 1e). A complete list of the 38 VOCs measured, their relative emission (%) and their respective Kovats retention indexes (RIs) is shown in Table A1 (Appendix A).

**Figure 2:**
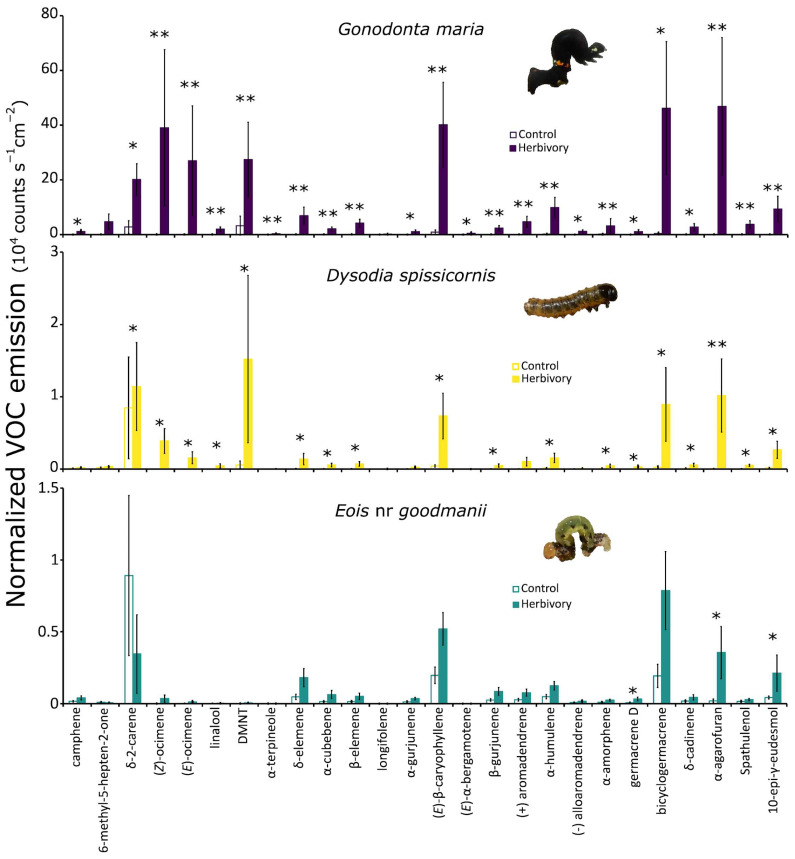
Changes in relative emission of individual volatiles in *P. arboreum* plants attacked by three different Lepidopteran herbivores in the field. Bars represent mean ± standard error of the relative emission (peak area) normalized by leaf area (10^4^ counts s^−1^ cm^−2^) for single compounds that were shown to be significant in the Random Forest analysis. Full bars represent leaves with actively feeding caterpillars of *Gonodonta maria*, *Dysodia spissicornis* and *Eois* nr *goodmanii* (“Herbivory”) while open bars represent respective control leaves (without herbivores). DMNT = (*E*)-4,8-dimethyl-1,3,7-nonatriene. * = *p* < 0.05 and ** = *p* < 0.001 for the comparison between herbivore-damaged leaves and their respective control (linear mixed model and estimated marginal means [emmeans]). For each herbivore species, bars are means ± standard error, *n* = 7 leaves with herbivores and *n* = 7 paired control leaves without herbivores, using branches of the same individual plant.

Random Forest analysis suggested that 28 out of the 39 VOCs measured by headspace sampling with PDMS tubing are responsible for the differences observed in VOC profiles of *P. arboreum* leaves with herbivory by the three different Lepidopteran herbivores compared to undamaged control leaves. The average emission for each of these 28 VOCs in *P. arboreum* leaves detected after herbivory by *Gonodonta*, *Dysodia* and *Eois* compared to their respective undamaged controls are shown in Figure 2 and Table A1 (Appendix A). Overall, herbivory increased the emission of individual VOCs by *P. arboreum* leaves compared to undamaged control leaves, which had low basal emission of most compounds (Figure 2, Linear Mixed Effects Models; * = *p* < 0.05, ** = *p* < 0.001, post hoc estimated marginal means contrasts within groups). All plants emitted blends composed of monoterpenes and sesquiterpenes, and the relative abundances of individual compounds varied among leaves damaged, depending on the herbivore identity. For example, leaves with herbivory by *Gonodonta* released higher amounts of the monoterpenes (*Z*)-β-ocimene and (*E*)-β-ocimene, while the homoterpene (*E*)-4,8-dimethyl-1,3,7-nonatriene (DMNT) was highly induced by both *Dysodia* and *Gonodonta* herbivory, but not in leaves damaged by *Eois* herbivory (Figure 2). Leaves subjected to *Gonodonta* herbivory exhibited the highest emission rates of individual VOCs, as well as the highest number of significantly induced single volatiles (24 compounds). Leaves attacked by *Dysodia* exhibited a significant increase in the emission of 18 single VOCs. In contrast, leaves damaged by the specialist *Eois* showed only a modest trend for increased VOC emission, and significant effects were only observed for three single VOCs: the sesquiterpenes germacrene D, α-agarofuran and 10-epi-γ-eudesmol. Separate analysis using only the undamaged control leaves showed no significant difference between the VOC profiles of undamaged leaves, despite differences in leaf age or season (Figure S1, NMDS stress = 0.054, PERMANOVA *p* = 0.325), while the comparison of only herbivore-induced VOC profiles showed significant differences caused by herbivore species (Figure S1, NMDS stress = 0.027, PERMANOVA *p* = 0.001). Analysis of the effect of herbivory on *P. arboreum* volatile profiles was significant for all three herbivore species (Figure S2, *Gonodonta*: NMDS stress = 0.013, PERMANOVA *p* = 0.004; *Dysodia*: NMDS stress = 0.031, PERMANOVA *p* = 0.045; *Eois*: NMDS stress = 0.028, PERMANOVA *p* = 0.005).

### 2.2. Generalist Herbivores Significantly Increase Total Volatile Release, but Total Emission Is Not Directly Proportional to Leaf Area Loss

**Figure 3:**
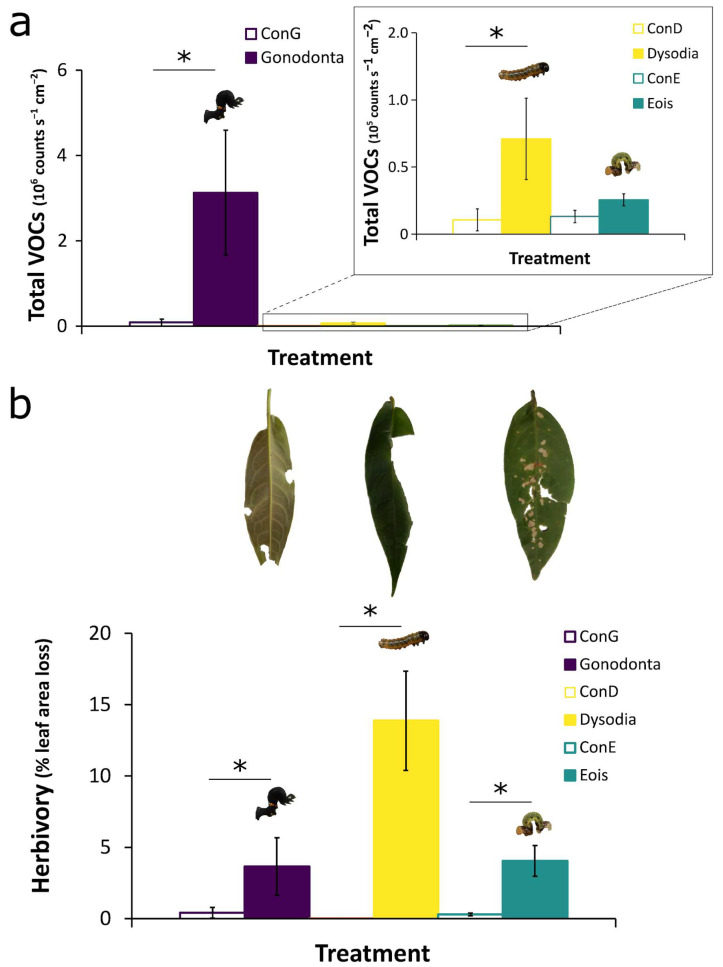
Both generalist herbivores significantly increase total volatile release by *P. arboreum* leaves, but emission is not directly proportional to leaf area loss (% herbivory). (**a**) Total volatile release in *P. arboreum* leaves after herbivory by *G. maria* (*Gonodonta*), *D. spissicornis* (*Dysodia*) and *Eois* nr *goodmanii* (*Eois*) and their respective undamaged control leaves (“ConG”, “ConD” and “ConE”). Total volatile release was calculated as the sum of the relative emissions (counts*s^−1^cm^−2^) of single compounds in each sample. Bars represent mean ± standard error, *n* = 7 leaves with herbivores and *n* = 7 paired control leaves without herbivores, for each herbivore species. * = *p* < 0.05 post hoc Tukey HSD comparisons of emmeans. (**b**) Herbivory expressed as % leaf area loss calculated based on photographs of *P. arboreum* leaves after VOC sampling for the same treatment groups as in (**a**). For each herbivore species, bars represent mean ± standard error, *n* = 7 leaves with herbivores and *n* = 7 paired control leaves without herbivores using branches of the same individual plant. * = *p* < 0.05, within groups emmeans contrasts, arcsine transformed data. Photographs of leaves above each herbivore show representative examples of the types of feeding damage caused by each lepidopteran species. *G. maria* feeding usually occurs on leaf edges, *D. spissicornis* is a leaf-rolling caterpillar that feeds on tissue within the rolled leaf and *E.* nr *goodmanii* feeds by scraping the leaf surface and leaving characteristic brown “windows” on *P. arboreum* leaves.

The total amount of VOCs released by *P. arboreum* leaves was quantified as the sum of all compounds released per sample. The increase in VOC emission was significant for plants with herbivory by *Gonodonta* and *Dysodia* compared to their respective controls (ConG and ConD, Figure 3a), but not for plants with *Eois* herbivory compared to its respective controls (ConE, Figure 3a; Total VOCs *Gonodonta*, ConG: *p* < 0.001; *Dysodia*, ConD: *p* = 0.05; *Eois*, and ConE: *p* = 0.77; post hoc pairwise comparisons of emmeans with Tukey HSD adjustment for multiple contrasts). This suggests that this specialist herbivore does not induce a substantial increase in total volatile emission in *P. arboreum*, despite the observed induction of a few individual volatile compounds such as (Z)-β-ocimene, (*E*)-β-ocimene, allo-ocimene, bicyclogermacrene and α-agarofuran (Figure 2). There were no significant differences between total VOC emissions by undamaged leaves in all three control treatments, even though samples were collected from leaves of different ages and harvested in different seasons (*p* > 0.50, post hoc for all pairwise comparisons of emmeans with Tukey HSD adjustment for multiple contrasts). This is likely due to the low basal emissions of VOCs by undamaged control leaves.

Since *P. arboreum* is a perennial plant species with relatively low leaf turnover, and samples were collected from naturally occurring individuals in the field, some control leaves that lacked actively feeding herbivores at the time of VOC collection displayed evidence of previous herbivore damage. Therefore, leaf area loss to herbivory (% Herbivory) in control samples was higher than 0 for all control groups. Nevertheless, leaf area loss (expressed as % Herbivory) in leaves damaged by actively feeding larvae was significantly higher than in corresponding controls for all three herbivore species (Figure 3b, Linear Mixed Effects Model with arcsine transformed data, Herbivory: *p* < 0.001; *Gonodonta*, ConG: *p* = 0.013; *Dysodia*, ConD: *p* < 0.001; *Eois*, ConE: *p* = 0.004; within groups emmeans contrasts for Herbivory|HerbivoreID). There was a non-significant trend toward differences in leaf area loss among herbivore species, and a significant interaction between herbivore species and herbivory on leaf area loss (Figure 3b, Linear Mixed Effects Model, Herbivory: *p* < 0.001, HerbivoreID: *p* = 0.086 and Herbivory*HerbivoreID: *p* < 0.001). This may be due to *Dysodia* larvae, which caused the greatest leaf area loss. Interestingly, total VOC emission in *P. arboreum* does not appear to be directly correlated to the extent of herbivore damage but rather to the identity of the herbivore. After adjusting for the proportion of leaf area lost and normalizing the volatile counts by the leaf area remaining at the end of the herbivory treatment, the total amount of VOC emission did not differ between leaves damaged by *Dysodia* and *Eois* feeding but was significantly higher in leaves with *Gonodonta* herbivory compared to those damaged by other herbivores (ANCOVA, Proportion Herbivory: *p* = 0.020 and Fisher’s LSD post hoc with Bonferroni Adjustment).

### 2.3. Herbivory Leads to Greater Changes in Effective Chemical Diversity of Volatiles than Leaf Age and Season

To investigate whether VOCs change measured in field-grown plants with naturally feeding herbivores could be influenced by leaf developmental stage or seasonal variation, we analyzed the essential oil extracts of pooled young and mature *P. arboreum* leaves lacking actively feeding herbivores. Leaf samples of *P. arboreum* for essential oil extraction were collected during both rainy (March) and dry (June) seasons at REBIO Mogi-Guaçu. Essential oil leaves were collected on the same dates that the volatile headspace of plants with and without herbivore feeding were sampled using PDMS tubes, although essential oil samples were taken from different plant individuals. Essential oil extraction was carried out by hydro-distillation of pooled leaves, and separate oils were extracted from young leaves (not fully expanded and have a reddish color) and from mature (fully expanded leaves in each season (*n* = 6 plants/season) (see Section 4.3 below for further details on sample collection and analysis)). Analysis by GC-MS revealed 46 different VOCs across all essential oil samples, of which 28 were also found in volatile headspace samples collected with PDMS in the field, and 18 were unique to essential oil samples. Table A1 and Table A2 (Appendix A) list all VOCs found in the PDMS headspace samples and essential oil samples, respectively, with their calculated Kovats Retention Indexes and relative concentrations (% total VOC content). The compound names shown in bold in Table A1 were detected exclusively in PDMS headspace samples (10 VOCs), while the names in Table A2 were detected exclusively in essential oil samples (18 VOCs).

The PDMS method was also chosen to evaluate if essential oil extractions are representative of the VOCs released by *P. arboreum* during ecological interactions. As the essential oil extraction method requires pooling of leaves from different individual plants to achieve sufficient mass for distillation, the results shown below represent a bulk sample for each of the following leaf stages and seasons: young leaves collected in March (rainy season), young leaves collected in June (dry season), mature leaves collected in March (rainy season) and mature leaves collected in June (dry season). Bulk essential oil samples were not used for multivariate analyses (NMDS and PERMANOVA), but changes in the complexity of the VOC blends due to leaf age and season were characterized using diversity partitioning measures to calculate the effective chemical diversity (^q^D) of these samples across different diversity orders (q), as a measure of volatile compositional diversity [24]. Diversity order (q) is a parameter that represents the relative contribution of high and low abundant compounds to effective chemical diversity, ranging from equal weighting (q = 0, which corresponds to compound richness) to weighting of individual compounds according to their relative abundances (q = 1, equivalent to the Hill numbers [25,26] for the Shannon–Wiener diversity index) and with increasing weight of high abundant compounds at q > 1. For comparison with bulk essential oil samples, the effective chemical diversity and diversity profile plots of headspace samples of *P. arboreum* with and without herbivory by the three native lepidopteran herbivores collected using PDMS were calculated using the mean values of VOCs for each group in the field experiment in Section 2.1 (*Gonodonta*, *Dysodia* and *Eois* herbivore damaged samples, and their respective undamaged controls ConG, ConD and ConE).

**Figure 4:**
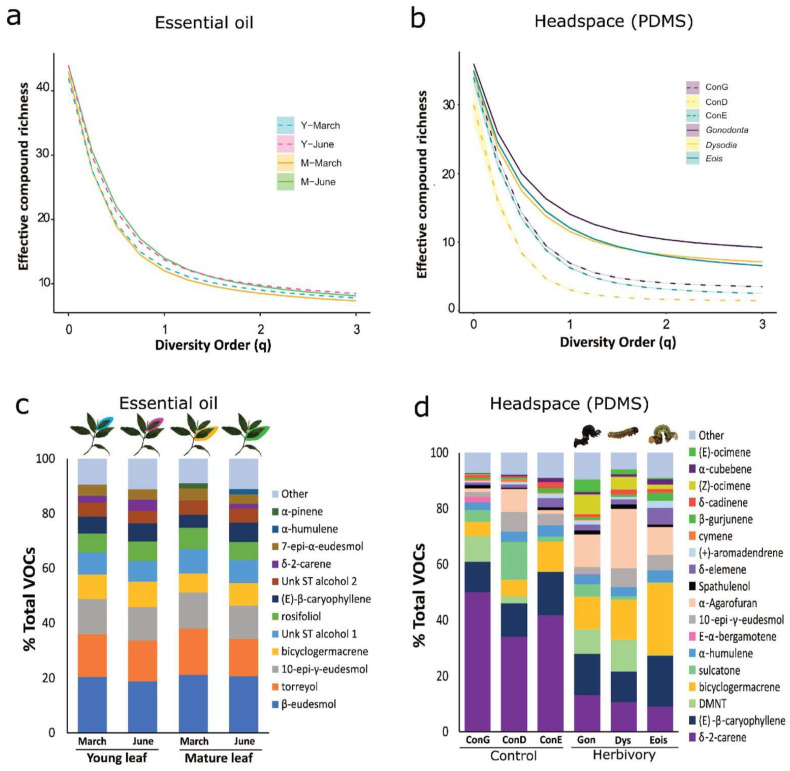
Complexity of *P. arboreum* volatiles shown as diversity profile plots and relative contribution of VOCs (% Total VOCs) for essential oils of young and mature undamaged leaves extracted in the rainy and dry seasons (**a**,**c**) and for headspace (PDMS) samples of leaves with herbivory by three native herbivores compared to undamaged controls (**b**,**d**). Diversity profile plots show the effective compound richness (or effective chemical diversity, ^q^D) at different diversity orders (q) for (**a**) essential oil samples of young (Y, dashed lines) and mature (M, full lines) *P. arboreum* leaves harvested from natural field-grown plants in March and in June 2018. (**b**) Diversity profile plots of headspace PDMS samples from leaves after herbivory by *Gonodonta*, *Dysodia* and *Eois* (full lines) and their respective undamaged control leaves (ConG, ConD and ConE, dashed lines). Diversity order (q) represents the relative contribution of high- and low-abundant compounds in a sample, ranging from equal weights (q = 0, equivalent to compound richness), where only the presence or absence of the compound is considered, to increased weight of high-abundant compounds at higher orders. At q = 1, each compound is weighted by its relative abundance (corresponding to the exponential of Shannon diversity). Shaded 95% confidence intervals were calculated via hierarchical bootstrapping. (**c**) Relative contribution (%) of VOCs in essential oils of *P. arboreum* leaves. Pictures above the graph show the type of leaf harvested in each group: young leaves harvested in March (blue) and in June (pink), and mature leaves harvested in March (yellow) and in June (green). (**d**) Relative contribution (%) of VOCs in the headspace profile of *P. arboreum* leaves after herbivory by *G. maria* (*Gonodonta*), *D. spissicornis* (*Dysodia*) and *Eois* nr *goodmannii* (*Eois*), and their respective undamaged control leaves (ConG, ConD and ConE). The same compound color key was used for (**c**,**d**) to highlight common VOCs, “Other” = sum of all VOCs < 1% relative abundance.

Diversity profile plots of the essential oil samples show that compound richness is similar in all oil samples (q = 0, VOC richness = 42 for young leaves in March and VOC richness = 44 for all other essential oils). However, at higher diversity order values (q > 0), a sharp drop in effective compound richness (^q^D) can be observed, indicating a high unevenness in VOC diversity, with the presence of a few high-abundant compounds and many low-abundant compounds (Figure 4a). These results also show that volatile chemical complexity was slightly higher in essential oil samples collected in the dry season (June), with the highest values observed for mature leaves. When individual VOCs are weighted by their relative abundance (q = 1) all four essential oils showed an effective compound richness (^1^D) between 12.02 and 14.02, with the highest diversities observed in the dry season samples (young leaves in March: ^1^D = 12.55 [95% confidence interval (CI) 12.54–12.57]; mature leaves in March: ^1^D = 12.02 [95% CI 12.01–12.03]; young leaves in June: ^1^D = 13.74 [95% CI 13.72–13.75]; and mature leaves in June: ^1^D = 14.02 [95% CI 14.01–14.04]). The analysis of volatile complexity in the headspace (PDMS) of *P. arboreum* leaves shows that herbivory by *Gonodonta*, *Dysodia* and *Eois* larvae increases effective chemical diversity compared to their respective controls (ConG, ConD and ConE) and is shown in the diversity profile plots in Figure 4b. Although compound richness (q = 0) is lower in headspace (PDMS) samples compared to essential oils, ranging from 30 to 36 compounds, the diversity profile plots also show a similar sharp decrease in ^q^D with increasing values of q. This indicates a high unevenness in VOC diversity, with the presence of a few high-abundant compounds and many low-abundant compounds. Herbivory by all three native lepidopteran herbivores caused a 2.0- to 3.8-fold increase in effective compound richness at q = 1 (Hill numbers for the Shannon–Wiener diversity index) compared to their respective controls (*Gonodonta*: ^1^D = 14.06 [95% CI 14.05–14.08]; ConG: ^1^D = 6.92 [95% CI 6.85–6.99]; *Dysodia*: ^1^D = 11.53 [95% CI 11.43–11.63]; ConD: ^1^D = 3.04 [95% CI 2.95–3.13]; *Eois*: ^1^D = 12.06 [95% CI 11.92–12.20]; and ConE: ^1^D = 6.26 [95% CI 6.12–6.40]).

The results of the diversity profile plots for essential oils of *P. arboreum* (Figure 4a) are supported by the relative distribution of the most abundant compounds across seasons and leaf developmental stages. The 12 most abundant compounds displayed similar distribution and contributed for 90.5%, 88.8%, 91% and 87% of the total VOC content, in young leaves harvested in March, young leaves in June, mature leaves in March and mature leaves in June, respectively. Figure 4c shows the relative amounts of volatiles (% total VOCs) in essential oil samples and highlights the contributions of the most abundant peaks (peaks > 1% of total VOC content). The changes in diversity profile plots for volatiles in the headspace of *P. arboreum* leaves with herbivory compared to undamaged controls are supported by the relative distribution of the most abundant compounds in these groups (Figure 4b,d). In this case, the 18 most abundant compounds (compounds > 1% total VOC profile) account for 90.40–94.13% of the total VOC content (ConG: 92.85%; ConD: 92.14%; ConE: 90.98%; *Gonodonta*: 90.40%; *Dysodia*: 94.13%; and *Eois*: 91.08%). While the three types of control samples are dominated by two major compounds, δ-2-carene and (*E*)-β-caryophyllene, which account for 45.98–60.87% of their total VOC content, these same compounds account for just 21.63–27.86% of VOCs from herbivore-damaged leaves. Thus, herbivory increases the diversity of *P. arboreum* leaf VOC profile compared to undamaged controls in addition to the increase in total VOC emission (Figure 2 and Figure 3a).

As seen in the analysis of compositional changes in volatiles by NMDS and PERMANOVA (Figure 1e) and for single compounds (Figure 2) above, herbivore-damaged leaves also differed in the identity of some of the most abundant compounds in their profile, such as the homoterpene DMNT, which represented 8.80% and 11.28% of total VOCs in *Gonodonta*- and *Dysodia*-damaged leaves, respectively, but only 0.21% in *Eois*-damaged samples. Also, the sesquiterpene bicyclogermacrene, which was the most abundant VOC and represented 25.87% of the total VOC content of *Eois*-damaged samples, was present in lower relative amounts of 11.70% and 14.33% in *Gonodonta* and *Dysodia*-damaged plants, respectively. Finally, only 4 out of 28 common compounds were among the most abundant VOCs in both PDMS headspace and essential oil samples, such as the monoterpene δ-2-carene and the sesquiterpenes (*E*)-β-caryophyllene, bicyclogermacrene and 10-epi-γ-eudesmol, highlighting that the differences between PDMS headspace samples and essential oil samples are not just due to the presence or absence of specific VOCs, but also to the relative amounts of common compounds in each blend (Figure 4c,d).

## 3. Discussion

Our results show that herbivory by three different naturally occurring lepidopteran caterpillars induces significant changes in the leaf volatile profile of a neotropical shrub. *P. arboreum* is common in the understory of tropical forests (Figure 1e). We also demonstrate that volatile headspace sampling using PDMS tubing provides a convenient way to collect volatile compounds in real time in the field, as previously shown for other plant species such as wild tobacco [23,27]. The exposure time (48 h) was chosen based on previous field trials with *Eois* sp. caterpillars on different *Piper* species (Stanton M.A., unpublished) and to standardize a minimum amount of feeding damage in each sample, in particular for the smaller specialist caterpillar species. This approach aims to reduce variability arising from differences in % herbivory among individual samples and ensures a quantifiable VOCs signal in GC-MS analysis. Moreover, this sampling method enables measurements of VOCs released in the plant headspace in a more ecologically realistic context than bulk volatile extraction traditionally used in phytochemistry techniques, such as hydro-distillation or analysis of VOCs from leaves that have been excised from the plant and measured or extracted later. Since the release of VOCs into the atmosphere upon herbivore damage can be both a rapid and transient response [4,12], it is possible that previous studies in this system have underestimated *Piper* VOC diversity. This may have occurred because of not controlling the timing of herbivory on leaves used for sample collection, missing the transient peak of VOC biosynthesis and/or release, or because analyses were conducted on undamaged leaves that had not yet induced the biosynthesis of herbivore-specific VOCs [3].

Undamaged *P. arboreum* leaves release low quantities of VOCs into the atmosphere, which hinder comparisons of volatile profiles of undamaged leaves at different ages and seasons using headspace sampling with PDMS tubes. In addition, given that the herbivores used are not commercially available, herbivory treatment could only occur during each herbivore species’ natural window of occurrence in nature. Therefore, factorial experimental designs testing for season, herbivore, and leaf age could not be performed. However, in the present study, the analysis of the VOC profiles of undamaged leaves using PDMS tubing showed that undamaged leaves did not differ between control groups, even when leaves were collected in different seasons (dry vs. rainy season) or when comparing leaves with different ages (young/not fully expanded vs. mature/fully expanded leaves). It is possible that the lack of separation among VOC profiles from undamaged control leaves (Figure S1) results from their significantly lower emission of single and total VOCs in these samples (Figure 2 and Figure 3). This reduced emission may have affected the ability to discriminate between these samples simply due to the instrument detection thresholds for measuring VOCs at very low concentrations [28]. Regardless of the possibility of limited detection of low basal VOC emission by undamaged samples, *P. arboreum* leaves with actively feeding herbivores consistently emitted higher amounts of single and total volatiles compared to undamaged control leaves sampled under the same conditions. This demonstrates the significant effect of herbivory on quantitative changes in VOC emission, causing additional qualitative changes in VOC blends (Figure 1e, Figure 2, Figure 3 and Figure S2).

Plant volatile emission has been shown to be context-dependent, and variation in volatile release can be caused by plant-based specificity (e.g., plant genotype) and herbivore-based specificity, which includes feeding guild and diet breadth, and can be influenced by abiotic factors such as temperature and precipitation (reviewed in [29]). It is possible that species-specific differences in caterpillar oral secretions (OSs) from the three herbivore species studied here may cause differences in VOC profiles of *P. arboreum* leaves after herbivory (Figure S2). This has been shown to be the case for elicitors present in caterpillar OSs, such as inceptin and volicitin, that change the blends of VOCs released by plants compared to those induced by mechanical damage or to other species, by inducing or suppressing different phytohormone pathways that affect VOC biosynthesis [30,31]. The two generalist herbivores also induced a higher number of single VOCs and of total VOC emissions by *P. arboreum* when compared to herbivory by the specialist caterpillar (Figure 2 and Figure 3a). While this contrasts with the pattern reported in a meta-analysis showing higher VOC inducibility in plants attacked by specialist herbivores [32], most of the generalist species in that analysis were piercing–sucking herbivores, which typically activate the salicylic acid pathway and reduce volatile emissions. Some specialist insects, such as mites, herbivores co-opting plant defenses, whitefly, etc., have been shown to suppress VOC emission by plants by repressing jasmonic acid (JA) signaling (reviewed in [33]), although chewing insects generally cause increased VOC emissions by activating the JA pathway. Our results are consistent with a study of native Dutch populations of *Brassica rapa* with 10 naturally occurring herbivores that varied in feeding guild and diet breadth, in which a higher induction of VOCs was observed after feeding by generalist herbivores compared to specialists [34]. These differences were apparent even when effects of diet breadth on VOCs were compared within chewing and piercing-sucking feeding guilds. Since generalist caterpillar species are frequently less abundant at the local plant or population scale but have higher occupancy rates at the landscape scale [35], it is possible that differences in plant VOC inducibility after generalist and specialist herbivory change the attraction of their respective predators and parasitoids at these different scales. Structural and compositional phytochemical diversity of *P. amalago* has been shown to have different effects on herbivores [36], but this study was carried out with NMR and HPLC analysis of non-volatile chemical defenses. Future studies comparing the effects of herbivore-specific VOC emission at different scales will help further our understanding of how VOCs mediate these ecological interactions in natural ecosystems.

Predators and parasitoids can use specific VOC blends as short-range host cues after locating the caterpillar’s host plant and can either respond innately to some herbivore-induced VOCs or learn to associate these cues with herbivore presence [10,37,38]. Many of the VOCs shown to be informative in the Random Forest analysis of herbivore-damaged leaves compared to undamaged leaves include terpenoids that have been previously shown to attract parasitoids, such as (*E*)-β-ocimene, linalool, DMNT and (*E*)-β-caryophyllene [33]. In addition, (*E*)-β-ocimene, (*E*)-β-caryophyllene and linalool were shown to drive responses of natural enemies of herbivores, such as *Adalia bipunctata*, *Chrysoperla* carnea, or entomopapathogenic nematodes [39,40]. Considering that their narrow diet breadth can limit specialist herbivores to just a few host plant species in the field, it is possible that specialist herbivores with limited mobility may benefit more than generalists from reduced induction of plant VOCs, to escape predators and parasitoids. Previous studies have shown that parasitoids are the main source of mortality for *Eois* sp. caterpillars [16,17] and a possible cause for the changes in seasonal distribution of these herbivores in Brazilian tropical forests [18,19]. This suggests that there could be a stronger selective pressure on *Eois* sp. caterpillar traits that affect plant VOC emission that attract parasitoids, but this hypothesis needs further testing. Alternatively, the lower total VOC emission observed in *P. arboreum* after *Eois* herbivory could reflect the lower leaf area loss caused by this smaller herbivore, compared to larger generalist caterpillars. The ANCOVA analysis suggests that after correcting for the proportion of leaf area loss, total VOC emission was similar between leaves damaged by *Eois* and *Dysodia*, but overall higher in *Gonodonta*-damaged leaves. These differences could be explained by the fact that *Gonodonta* larvae feed preferentially on young leaves of *P. arboreum*, unlike the other two herbivores. Many plants have higher VOC biosynthesis rates in young leaves compared to fully mature leaves [41], and it is possible that this accounts for the higher total VOC released in leaves of the *Gonodonta* treatment (Figure 3a), at approximately 100 times the rate. However, apart from quantitative differences, qualitative differences in blend composition were observed between leaves damaged by all three herbivores (Figure 1e and Figure 2). Furthermore, these herbivores significantly altered the VOC profile of *P. arboreum* leaves compared to undamaged controls (Figure S2), even herbivory by the specialist *Eois* that significantly induced fewer individual compounds.

From the plant’s perspective, increased and specific VOC emissions are favorable if they also increase the attraction of natural enemies of their herbivores, acting as an indirect defense. However, there are many cases of specialist herbivores co-opting plant defenses for their own benefit, including VOCs to find their host plants [11,42,43,44]. In this context, an increase in phytochemical diversity of volatiles could benefit the host plant by disrupting host plant finding by specialist herbivores. A study on *Piper* communities in Costa Rica has suggested that reduced specialist herbivory by *Eois* caterpillars is linked to increased diversity of plant volatile chemistry [5], although in this case, volatile diversity was characterized by extracting leaves with solvents and not with headspace volatile sampling. Another study suggested that decreased volatile diversity in *Piper* communities, measured by analysis of field-collected leaves without actively feeding herbivores, is related to higher herbivory in the field [3]. Our results show that herbivory by all three herbivore species increases the compositional diversity of *P. arboreum* VOC profiles (Figure 4) [24], leading to a 2.0- to 3.8-fold increase in the effective compound richness. The diversity profile plots and analysis of the most abundant VOCs in the headspace (PDMS) samples also revealed a high unevenness of volatile composition, characterized by the presence of a few high-abundant compounds and many low-abundant compounds in each profile (Figure 4b,d). Analysis of compound richness of essential oils from undamaged leaves that were harvested at different ages and seasons also revealed a high unevenness in volatile composition (Figure 4a,c). Essential oils from young and mature *P. arboreum* leaves collected in June had a slightly higher chemical diversity (^1^D) than oils from leaves of both ages collected in March, suggesting there may be some seasonal differences in standing VOC pools within the leaves. Overall, the chemical diversity of essential oils (^1^D between 12.02 and 14.02) was similar to that of herbivore-damaged headspace samples collected by PDMS (^1^D between 11.53 and 14.06), but analysis of the most abundant compounds revealed differences in the composition of essential oils and headspace VOC profiles. Since our essential oils were extracted from bulk leaf samples without replication, we did not perform multivariate analysis on these samples, but the analysis of functional chemical diversity and differences in composition of the most abundant VOCs compared to headspace samples collected by PDMS highlights the differences resulting from different volatile sampling methods in this system (Figure 4). This suggests that the composition of volatiles within leaves may differ from the blend of volatiles released into the atmosphere during a plant’s interactions. While the extraction of essential oils is valuable to obtain large amounts of VOCs from plant tissues, and was used in this study to characterize the standing pools of VOCs within leaves, the harsh extraction conditions likely extract compounds that would not usually be released into the atmosphere under natural conditions. In addition, structures of some compounds are prone to thermal rearrangements [8]. Therefore, we consider the sampling method with PDMS tubing to be more adequate for studying ecological functions of plant volatiles, since it more closely mimics the natural conditions under which these VOCs are released in nature and allows for easy sampling of individual plants or plant parts.

## 4. Materials and Methods

### 4.1. Study System

The study was carried out in a protected area situated in a transition zone between the Cerrado savannah and Atlantic Forest in southeastern Brazil, at the Reserva Biológica de Mogi-Guaçu (REBIO Mogi-Guaçu, São Paulo state, Brazil). The *P. arboreum* plants were growing naturally within the area known as “Gleba A” of the REBIO Mogi-Guaçu, which is characterized as an area of forested savannah (“Cerradão” vegetation) cut by a small stream [45]. Individual plants selected for volatile headspace collections or for essential oil extraction were inspected for the presence or absence of feeding herbivores, and were growing at least 2 m apart from other replicates. Plants and insects were collected under permit numbers 89239-6 (Sisbio). Caterpillars were reared in individual 300 mL clear plastic pots with lids, assigned a collection code, and provided with leaves of the same *Piper* species from which they were collected as a food source until pupation. Pupae were placed in new 150 mL clear plastic containers with lids with a moist cotton ball to maintain a humid environment for pupal development and monitored for 30 days for the emergence of adults or parasitoids. All insects were kept in a room at the University of São Paulo (USP) under constant temperature (24 °C) and a 12 h light–12 h dark cycle. Emerging Lepidoptera adults were photographed and stored at −20 °C for morphological and molecular identification. Any parasitoids emerging from larvae or pupae were also photographed and stored in ethanol for identification. The insect vouchers were deposited at the Museu de Biodiversidade, Universidade Estadual de Campinas (UNICAMP) in Campinas, SP, Brazil.

### 4.2. Field Volatile Headspace Collection Using Polydimethysiloxane (PDMS)

Volatiles were collected at the field site in the REBIO Mogi-Guaçu during two different seasons, corresponding to the peaks in caterpillar abundance for the studied species, in the summer/wet season for *G. maria* and *D. spissicornis*, and in the winter/dry season for *E.* nr *goodmanii*. Individual *P. arboreum* plants were inspected for the presence of caterpillars of either *G. maria* (hereafter, “*Gonodonta*” treatment) or *D. spissicornis* (hereafter, “*Dysodia*” treatment) in March 2018, and volatiles were collected from single leaves with actively feeding caterpillars on them. In June 2018, *P. arboreum* plants with *E.* nr *goodmanii* damage and actively feeding caterpillars (hereafter, “*Eois*” treatment) were sampled for volatiles in the same way. For each herbivore-damaged plant sampled, a single leaf without herbivores from an opposite branch on the same plant was chosen as a control in a paired analysis, to control for variation in volatile emission between individual plants and to obtain an equal number of replicates for each set of herbivore-damaged, undamaged control leaves (*n* = 7 leaves with herbivores and *n* = 7 control leaves without any herbivores/plant for each herbivore type, leading to a total of *n* = 14 plants in March and *n* = 7 plants in June). Control leaves were chosen to be at the same position on the branch (counting from the tip) as the paired herbivore-damaged leaves sampled on the same plant. Previous experiments with *Piper* sp. have shown that undamaged leaves from a neighboring branch show basal levels of volatile emission comparable to those of undamaged plants.

Each sampled leaf was enclosed in a 2 L custom-made bag of plastic baking foil (Qualitá, Alumileste Indústria e Comércio Ltd.a, Campo Limpo Paulista, SP, Brazil) with openings on two opposite sides, like a cylinder. The plastic bag was closed with twist-ties around the leaf petiole and the opposite open end to enclose the leaf and herbivores for volatile sampling, as shown in Figure 1d. Volatile sampling was carried out using a static headspace method using polydimethylsiloxane (PDMS) silicone tubing, which is very sensitive for sampling of mono- and sesquiterpenes, prepared in a method slightly modified from [23]. Briefly, PDMS tubing was cut to size and soaked overnight in 100% HPLC grade acetonitrile (Merck, Darmstadt, Germany), dried at 200 °C, and cooled down under a N_2_ flow and stored in amber glass vials under N_2_ atmosphere until use. For sampling, two pieces of PDMS silicone tubing (25 mm each) were placed on a 20 cm long piece of metal wire that was inserted in the bag and held in place by the twist-tie around the leaf petiole so as to keep the PDMS tubing suspended within the plastic foil bag, without touching the leaf or plastic surfaces. After 48 h exposure, the silicone tubes were placed in 2 mL glass vials with screw caps and stored in a freezer (−20 °C) until analysis by gas chromatography coupled with mass spectrometry (GC-MS). For each measurement period, a clean empty plastic bag was sampled as a blank of background volatiles and contaminants.

A single PDMS tube from each sample was subjected to thermal desorption and analyzed using a Shimadzu GCMS-QP2010, equipped with an Rtx-5ms column (length 30 m, ID 0.25 mm, film thickness 0.25 µm, Restek, Bellefonte, PA, USA) using helium as a carrier gas (4.5 mL/min), with a scan range of 35–400 *m*/*z* at 2500 spectra s^−1^. Silicon tubes were placed in 20 mL glass vials and thermally desorbed for 10 min at 180 °C using an AOC 5000 Plus auto-sampler (Shimadzu, Kyoto, Japan) equipped with a gas-tight 2.5 mL syringe and headspace analysis unit (HS-20, Shimadzu). Samples were injected at 200 °C (2000 µL, 1:5 split injection) and the oven program started at 40 °C for 1 min, increased at 5 °C min^−1^ to 165 °C, with a second ramp of 1 °C min^−1^ to 172 °C, and then at 26 °C min^−1^ to 280 °C, and was held for 1 min. Individual volatile compound peaks were identified using extracted ion traces of three specific ions and quantified by the peak area of the most abundant ion trace per compound using the GCMS Postrun Analysis software 2.21 (Shimadzu) and a custom-made analysis method. Peak area (count s^−1^) for individual compounds was normalized by leaf area (cm^2^) for all experiments. Contaminants and background peaks were excluded from data analysis by comparison of samples to the background control. After removal of background peaks, control and herbivore feeding samples were compared to search for herbivore-specific volatiles. The identification of compounds was conducted by calculating Kovats retention indexes (RIs) in relation to alkane standards, and comparison to RIs in available databases ([46] Pherobase, Pubchem), together with comparison of their mass spectra to those of Wiley version 9 (Wiley, Hoboken, NJ, USA) and NIST (National Institute of Standards and Technology, Gaithersburg, MD, USA) spectral libraries, and commercial standards, when available.

Immediately after volatile sampling, leaves were photographed to calculate leaf area and herbivory (% leaf area loss). Leaf area was estimated from black and white photographs of the sampled leaf against a standardized white background with a black 1 cm^2^ square for calibration, using the ImageJ software 1.x as described in [47] and correcting for area lost to herbivore damage. Larvae from the herbivore treatments were collected and brought to the lab at the Institute of Chemistry at the University of São Paulo, where they were reared on fresh *P. arboreum* leaves in individual 100 mL plastic containers with lids, under constant temperature (24 °C) and a 12 h light–12 h dark cycle until reaching the adult stage. Emerging adults were photographed and stored at −20 °C for morphological and molecular identification.

### 4.3. Essential Oil Extractions

To control for seasonal and leaf age differences in standing volatile pools within *P. arboreum* leaves that could account for differences in volatiles released in the leaf headspace collected with PDMS, a separate set of *P. arboreum* plants without actively feeding herbivores were harvested in March and June 2018 (*n* = 6 plants/season, young and mature leaves were harvested from the same individuals), and pooled samples of freshly harvested young and mature leaves from each sampling season were used for essential oil extractions to obtain leaf volatile pools. Briefly, plant tissue was kept fresh until extraction by transporting *P. arboreum* cuttings with humidified cotton wrapped around the stem in plastic bags to the lab, where they were stored for 24 h at 4 °C before extraction by hydro-distillation in a Clevenger-type apparatus using 1 L of distilled water in a 2 L round flask using a modified method [15]. Essential oil extracts were collected and stored in the freezer until analysis by GC-MS. Samples were analyzed using a Shimadzu GCMS-QP2010, equipped with an Rtx-5ms column (length 30 m, ID 0.25 mm, film thickness 0.25 µm, Restek) using helium as a carrier gas (4.5 mL/min), with a scan range of 35–400 *m*/*z* at 2500 spectra s^−1^. Essential oil extracts were diluted 1:1,000,000 in HPLC-grade ethyl acetate (Sigma-Aldrich, Saint Louis, MO, USA) and injected at 200 °C (1 µL, 1:20 split injection) using liquid injection in an AOC 5000 Plus auto-sampler (Shimadzu) equipped with a 10 µL syringe. Samples were separated using the same GC-MS oven program as for the PDMS samples above, and peak identification and integration were performed as described for PDMS samples. Individual peak areas were normalized as a % of the total, where the total volatile content of a sample was considered as the sum of all peak areas in that sample.

### 4.4. Statistical Analysis

Statistical analyses were performed using R (https://www.r-project.org/ accessed on 10 April 2022). Total VOC profiles were analyzed by non-metric multidimensional scaling (NMDS) to visualize compositional differences in plant profiles with and without herbivory (VEGAN package, *metaMDS* function, plotted with GGPLOT2 package). Differences in the overall composition of VOCs emitted by *P. arboreum* leaves with and without herbivory by one of the three lepidopteran species (*Gonodonta maria*, *Dysodia spissicornis* and *Eois* cf *hyperythraria*) were tested in a paired design using Permutational Analysis of Variance (PERMANOVA) grouped by herbivory and herbivore identity (ID), with the following levels for “herbivory”: undamaged control, herbivore damage; and for “herbivoreID”: *Gonodonta*, *Dysodia*, *Eois*. PERMANOVA analysis was carried out in the VEGAN package, *adonis* function with restricted permutations defined by the *how* function using individual plants as a random block [48]. We also used Random Forest, a bootstrapping algorithm that is robust to autocorrelations between predictor variables, to determine which individual VOCs are most likely to be responsible for the observed differences in profile between control and herbivore-damaged samples in the NMDS and PERMANOVA analysis (BORUTA package, functions *Boruta* and *TentativeRoughFix*) [49].

Differences in the relative emission between herbivore-damaged samples and respective controls for single volatile compounds that were identified as important in Random Forest analysis were analyzed using Linear Mixed Effects Models, with the individual plant as a random effect and with herbivory and herbivore identity (ID) as fixed effects (“herbivory” levels: undamaged control, herbivore damage; “herbivoreID” levels: *Gonodonta*, *Dysodia*, *Eois*; NLME package, *lme* function). Significant effects were tested with multiple comparisons using estimated marginal means (EMMEANS package, *emmeans* function for herbivory|herbivoreID or with *emmeans* for interactions herbivory*herbivoreID, “tukey adjust” for multiple comparisons). Data was log + 1 transformed when necessary. Total VOC emission (the sum of all VOCs in a sample) was log + 1 transformed, and leaf area loss to herbivory (% Herbivory) was arcsine transformed; both were analyzed using Linear Mixed Effects Models with the individual plant as a random effect and with herbivory and herbivore identity (ID) as fixed effects, as described above for single volatile compounds. The effect of the proportion of leaf area loss (covariate) and herbivoreID (grouping factor) on total VOC emission (ln transformed data) was analyzed by Analysis of Covariance (ANCOVA) using the BROOM, DPLYR and AGRICOLAE packages.

Diversity partitioning was carried out using the iNEXT. Three-dimensional [50] and GGPLOT2 packages to calculate functional chemical diversity (effective compound richness) for q = 0 to q = 3 with hierarchical bootstrapping for 95% confidence intervals and make diversity profile plots.

## Data Availability

Data Availability Statements are available in section “MDPI Research Data Policies” at https://www.mdpi.com/ethics (accessed on 15 January 2026).

## Supplementary Materials

The following supporting information can be downloaded at: https://www.mdpi.com/article/10.3390/plants15020290/s1, Figure S1. Non-metric multidimensional scaling (NMDS) of volatile blends released by Piper arboreum plants for all control leaves without herbivores (top panel) and for all leaves after herbivory by three different Lepidopteran herbivores (top panel) (n = 7 for each herbivore species). Full symbols represent leaves with herbivory by *Gonodonta maria* (“Gonodonta”), *Dysodia spissicornis* (“Dysodia”) and *Eois hyperythraria* (“Eois”). Open symbols represent respective undamaged control leaves for G. maria (“Con G”), *D. spissicornis* (“Con D”) and *E. hyperythraria* (“Con E”). 2D stress value for NMDS and *p*-values for the PERMANOVA analysis for all control samples (top panel) and all herbivore damaged samples (bottom panel) are shown in the upper right corner; Figure S2. Non-metric multidimensional scaling (NMDS) of volatile blends released by Piper arboreum leaves with herbivory by *Gonodonta maria* (“Gonodonta”) and for paired control leaves without herbivores (“Con G”) (top panel); for leaves after herbivory by *Dysodia spissicornis* (“Dysodia”) and its paired undamaged control leaves (“Con D”) (middle panel) and for *P. arboreum* leaves after herbivory by *Eois hyperythraria* (“Eois”) and its paired undamaged control leaves (“Con E”). 2D stress value for NMDS and *p*-values for the PERMANOVA analysis within each herbivore species and paired controls are shown in the upper right corner.

## Appendix A

**Table A1:** List of VOCs identified by GC-MS sampled using PDMS tubes from leaves of *P. arboreum* naturally occurring in the field with at least 48 h of herbivory by *Gonodonta maria* (*Gonodonta*), *Dysodia spissicornis* (*Dysodia*) and *Eois* nr *goodmanii* (*Eois*), and respective paired control leaves (ConG, ConD and ConE). Calculated Kovats Retention Index (RI) and RI values reported in the literature (RI lit) are shown. Values are average relative amounts (%) ± standard error. ^S^ = compound identity confirmed by synthetic standards. Non-annotated compounds were putatively identified based on similarities of RI and direct comparison with the mass spectra from libraries. DMNT = (*E*)-4,8-dimethyl-1,3,7-nonatriene. Compounds highlighted in gray were not considered significant in the Random Forest analysis, and compound names shown in bold font were obtained exclusively in PDMS samples and not in the essential oils (see text for further details).

Compound	RI	RI Lit	ConG		*Gonodonta*		ConD		*Dysodia*		ConE		*Eois*	
α-pinene ^S^	933	932	0.05	±0.03	0.03	±0.03	0.10	±0.10	-	-	0.56	±0.25	0.15	±0.08
**camphene ^S^**	949	946	2.58	±1.42	1.08	±0.77	4.47	±2.06	0.67	±0.62	1.90	±1.05	1.40	±0.28
β-pinene ^S^	977	974	-	-	-	-	0.02	±0.02	0.18	±0.19	0.01	±0.01	0.04	±0.02
**6-methyl-5-hepten-2-one**	986	981	4.24	±1.71	4.42	±3.91	13.59	±7.48	1.26	±0.72	1.91	±0.83	0.18	±0.05
α-phellandrene ^S^	1004	1002	0.50	±0.27	0.32	±0.15	0.18	±0.13	0.20	±0.12	0.10	±0.04	0.01	±0.01
δ-2-carene ^S^	1010	1008	49.99	±10.70	13.16	±5.19	33.98	±16.10	10.72	±3.05	41.77	±8.44	8.99	±5.43
cymene ^S^	1025	1020	0.49	±0.28	0.31	±0.18	1.10	±0.70	0.07	±0.05	0.19	±0.09	0.02	±0.01
limonene ^S^	1029	1024	0.20	±0.15	0.01	±0.01	0.11	±0.12	0.02	±0.02	0.03	±0.03	0.03	±0.01
(*Z*)-β-ocimene ^S^	1037	1032	0.24	±0.17	7.05	±2.74	-	-	4.55	±2.58	0.04	±0.03	1.66	±1.23
(*E*)-β-ocimene ^S^	1048	1044	0.16	±0.11	4.48	±1.94	0.03	±0.04	1.76	±1.02	0.01	±0.01	0.54	±0.39
linalool ^S^	1099	1095	0.33	±0.17	1.54	±0.84	0.42	±0.33	0.57	±0.25	0.09	±0.06	0.08	±0.04
DMNT ^S^	1115	1114	9.25	±5.85	8.80	±2.79	2.57	±1.40	11.28	±7.69	0.14	±0.08	0.21	±0.09
**allo-ocimene**	1129	1128	0.03	±0.02	1.03	±0.39	0.01	±0.02	0.68	±0.39	0.01	±0.01	0.30	±0.23
**α-terpineole ^S^**	1194	1186	0.17	±0.11	0.20	±0.11	0.00	±0.00	-	-	0.32	±0.19	0.03	±0.01
δ-elemene	1344	1335	0.86	±0.47	2.11	±0.48	1.16	±0.80	1.75	±0.48	3.22	±0.79	5.90	±0.46
α-cubebene	1356	1345	0.37	±0.23	0.92	±0.28	0.54	±0.36	1.01	±0.36	1.46	±0.31	1.90	±0.18
α-ylangene	1381	1373	0.09	±0.10	0.12	±0.08	-	-	0.03	±0.03	-	-	-	-
α-copaene ^S^	1385	1374	0.20	±0.14	0.47	±0.24	0.22	±0.19	0.34	±0.11	1.31	±0.73	1.04	±0.10
**β-bourbonene**	1396	1387	0.18	±0.14	-	-	-	-	-	-	0.10	±0.09	0.08	±0.07
β-elemene	1399	1389	0.83	±0.37	1.84	±0.47	0.74	±0.38	1.32	±0.34	0.97	±0.24	1.61	±0.24
**longifolene ^S^**	1422	1407	0.86	±0.34	0.09	±0.10	0.42	±0.30	0.02	±0.02	-	-	-	-
α-gurjunene ^S^	1422	1409	0.23	±0.21	0.31	±0.20	0.44	±0.31	0.44	±0.15	0.89	±0.21	1.23	±0.09
(*E*)-β-caryophyllene ^S^	1433	1417	10.88	±4.63	14.70	±3.02	12.00	±3.77	10.91	±1.54	15.47	±2.11	18.26	±1.34
**(*E*)-α-bergamotene ^S^**	1441	1432	1.94	±0.79	0.24	±0.26	-	-	-	-	-	-	-	-
β-gurjunene	1441	1431	0.56	±0.50	0.91	±0.31	0.49	±0.37	0.75	±0.21	1.76	±0.44	2.84	±0.22
aromadendrene ^S^	1453	1439	0.58	±0.52	1.43	±0.49	0.86	±0.90	0.83	±0.38	1.70	±0.43	2.56	±0.21
α-humulene ^S^	1468	1452	2.78	±0.98	3.62	±0.92	3.67	±0.91	3.29	±0.90	4.09	±0.68	4.34	±0.34
**alloaromadendrene ^S^**	1476	1458	0.43	±0.21	0.41	±0.16	0.02	±0.02	0.23	±0.16	0.74	±0.30	0.55	±0.13
α-amorphene	1485	1483	0.42	±0.26	1.41	±1.11	0.38	±0.23	0.57	±0.18	1.23	±0.45	0.80	±0.06
germacrene D	1493	1481	0.03	±0.03	0.24	±0.09	-	-	0.25	±0.12	0.32	±0.14	1.02	±0.07
β-selinene	1500	1490	-	-	0.07	±0.07	0.14	±0.16	0.07	±0.06	0.17	±0.09	0.37	±0.09
bicyclogermacrene	1509	1500	5.12	±2.94	11.70	±3.22	5.91	±4.08	14.33	±2.65	10.64	±3.79	25.87	±2.21
δ-amorphene	1524	1511	0.05	±0.03	0.06	±0.03	0.20	±0.16	0.03	±0.03	0.28	±0.12	0.11	±0.06
δ-cadinene	1531	1522	1.09	±0.29	1.03	±0.52	0.44	±0.25	1.85	±0.78	2.11	±0.62	1.32	±0.11
**(*E*)-iso-γ-bisabolene**	1542	1529	-	-	0.38	±0.27	-	-	0.26	±0.22	-	-	0.12	±0.10
**α-agarofuran**	1562	1548	1.28	±0.35	11.55	±2.79	8.23	±6.04	21.34	±4.39	1.32	±0.50	9.94	±2.20
spathulenol	1590	1577	1.27	±0.76	1.50	±0.58	0.52	±0.54	1.69	±0.85	1.07	±0.25	1.03	±0.19
10-epi-γ-eudesmol	1638	1622	1.75	±0.45	2.47	±0.47	7.05	±2.37	6.74	±1.82	4.08	±0.73	5.52	±0.96

**Table A2:** List of VOCs identified by GC-MS in the essential oils of young and mature leaves of *Piper arboreum* naturally occurring in the field, harvested in March (autumn) or June (winter), with calculated Retention Index (RI) and RI values reported in the literature (RI lit). Values are average relative amounts (%). ^S^ = compound identity confirmed by synthetic standards. Non-annotated compounds were putatively identified based on similarities of RI and direct comparison with the mass spectra from libraries. DMNT = (*E*)-4,8-dimethyl-1,3,7-nonatriene, and Unk ST = unknown sesquiterpene. Compounds shown in bold font were obtained exclusively in essential oil samples and not in headspace samples collected with PDMS tubes (see text for further details).

Compound	RI	RI Lit	March	June	March	June
α-pinene ^S^	933	932	0.06	0.49	1.85	0.40
β-pinene ^S^	977	974	-	0.10	0.36	0.08
**β-myrcene ^S^**	991	988	0.06	0.09	0.16	0.05
α-phellandrene ^S^	1004	1002	0.15	0.17	0.04	0.12
δ-2-carene ^S^	1010	1008	2.55	4.03	0.12	1.77
cymene ^S^	1026	1020	-	0.06	0.16	-
limonene ^S^	1030	1024	0.04	0.07	0.14	0.07
(*Z*)-β-ocimene ^S^	1039	1032	0.59	0.65	0.04	0.50
(*E*)-β-ocimene ^S^	1050	1044	0.19	0.20	-	0.17
**α-terpinolene ^S^**	1090	1086	0.07	0.07	0.02	0.05
linalool ^S^	1101	1095	0.08	0.11	0.03	0.07
DMNT ^S^	1118	1118	0.70	0.82	0.09	0.61
δ-elemene	1345	1335	0.05	0.06	0.04	0.09
α-cubebene	1357	1345	0.06	0.05	0.04	0.10
**cyclosativene**	1377	1369	0.04	0.05	0.02	0.09
α-ylangene	1383	1373	0.04	0.05	0.03	0.13
α-copaene ^S^	1386	1374	0.07	0.18	0.05	0.17
β-elemene	1400	1389	0.42	0.67	0.41	0.67
α-gurjunene ^S^	1421	1409	0.13	0.16	0.13	0.32
(*E*)-β-caryophyllene ^S^	1442	1417	6.21	6.70	4.77	7.16
β-gurjunene	1449	1431	0.59	0.67	0.50	0.85
aromadendrene ^S^	1459	1439	0.19	0.21	0.20	0.56
**α-himachalene**	1462	1449	0.05	0.08	-	0.11
***E*** **-β-farnesene ^S^**	1461	1454	0.19	-	0.22	0.26
α-humulene ^S^	1471	1452	1.70	1.98	1.46	1.95
α-amorphene	1489	1483	0.16	0.39	0.15	0.20
germacrene D	1500	1481	0.66	0.51	0.45	0.43
β-selinene	1505	1490	0.07	0.12	0.08	0.16
**α-selinene**	1506	1498	0.05	0.04	0.03	0.08
bicyclogermacrene	1530	1500	8.87	9.20	6.95	8.29
δ-amorphene	1529	1511	0.34	-	0.58	0.29
δ-cadinene	1522	1522	0.52	0.55	0.47	0.86
**(*E*)-cadina-1,4-diene**	1527	1533	-	0.09	-	0.14
**hedycaryol**	1566	1546	0.70	0.57	0.90	1.34
**(*E*)-nerolidol ^S^**	1573	1561	0.74	0.49	0.83	0.65
spathulenol	1592	1577	0.09	0.38	0.30	0.34
**viridiflorol**	1598	1592	0.31	0.41	0.33	0.51
guaiol	1606	1600	0.37	0.47	0.28	0.61
**rosifoliol**	1615	1600	6.81	7.03	7.98	6.50
10-epi-γ-eudesmol	1631	1622	12.85	12.19	13.16	12.11
**Unk ST alcohol 1**	1648	-	8.21	7.68	8.72	8.47
**torreyol**	1650	1644	15.48	14.87	16.91	13.52
**Unk ST alcohol 2**	1660	-	5.11	4.50	5.20	5.03
**β-eudesmol**	1660	1649	20.42	18.81	21.19	20.69
**7-epi-α-eudesmol**	1668	1662	4.01	3.80	4.36	3.43
**bulnesol**	1679	1671	-	0.16	0.21	-
