# Supplementary material for: Herbivore-Induced Specificity and Diversity in Piper arboreum Volatiles
Source: Plants (Basel). 2026 Jan 18;15(2):290. doi: 10.3390/plants15020290 (PMC12845114; doi:10.3390/plants15020290)
Supplement: Supplementary file 1 [file plants-15-00290-s001.zip › plants-3976629-supplementary.pdf]

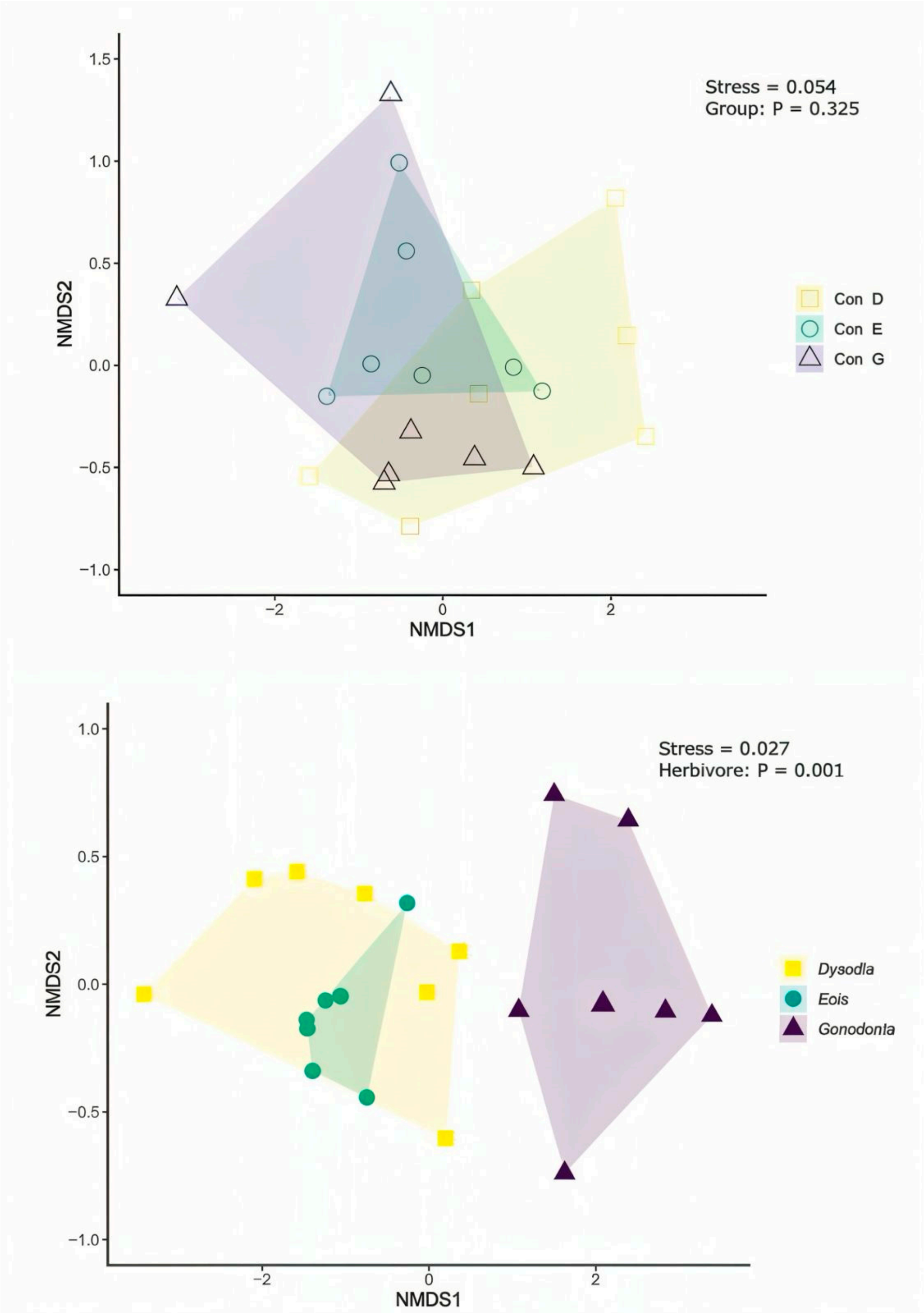

Figure S1. Non-metric multidimensional scaling (NMDS) of volatile blends released by *Piper arboreum* plants for all control leaves without herbivores (top panel) and for all leaves after herbivory by three different Lepidopteran herbivores (top panel) (n = 7 for each herbivore species). Full symbols represent leaves with herbivory by *Gonodonta maria* ("*Gonodonta*"), *Dysodia spissicornis* ("*Dysodia*") and *Eois hyperythraria* ("*Eois*"). Open symbols represent respective undamaged control leaves for *G. maria* ("Con G"), *D. spissicornis* ("Con D") and *E. hyperythraria* ("Con E"). 2D stress value for NMDS and P-values for the PERMANOVA analysis for all control samples (top panel) and all herbivore damaged samples (bottom panel) are shown in the upper right corner. .

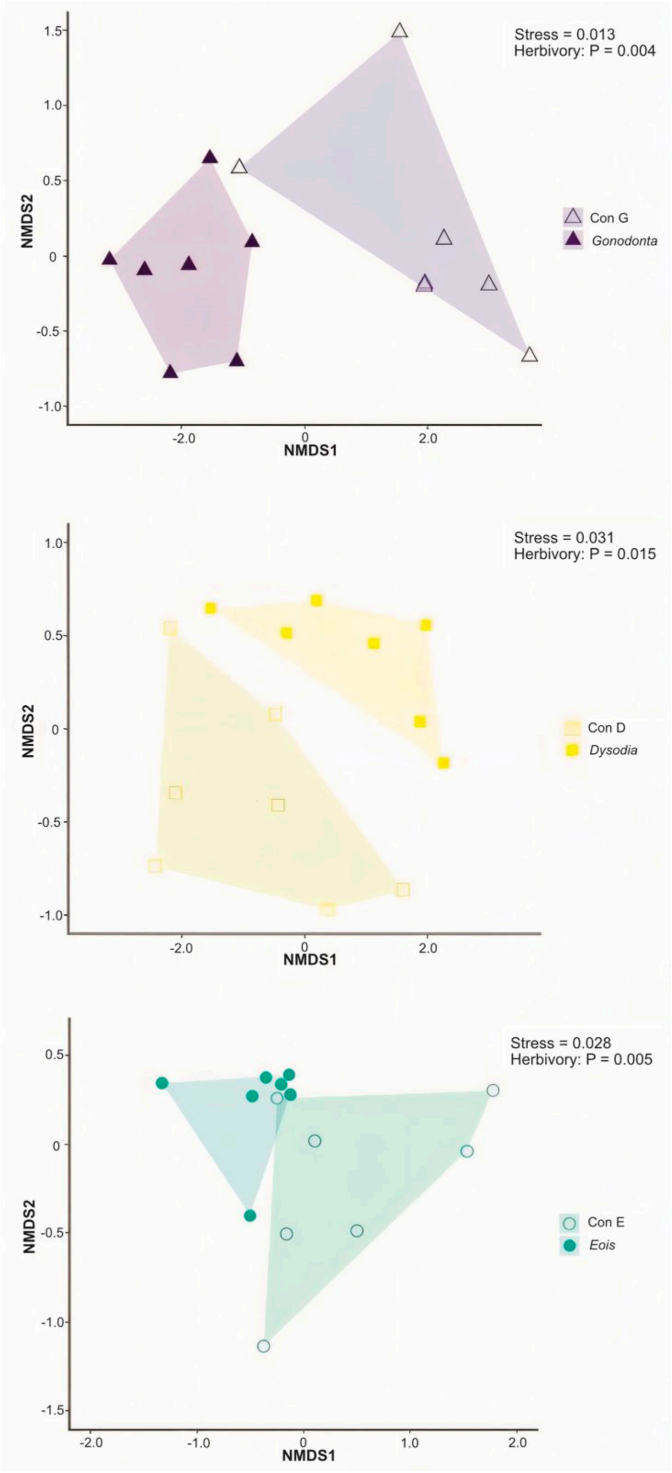

Figure S2. Non-metric multidimensional scaling (NMDS) of volatile blends released by *Piper arboreum* leaves with herbivory by *Gonodonta maria* ("*Gonodonta*") and for paired control leaves without herbivores ("Con G") (top panel); for leaves after herbivory by *Dysodia spissicornis* ("*Dysodia*") and its paired undamaged control leaves ("Con D") (middle panel) and for *P. arboreum* leaves after herbivory by *Eois hyperythraria* ("*Eois*") and its paired undamaged control leaves ("Con E"). 2D stress value for NMDS and P-values for the PERMANOVA analysis within each herbivore species and paired controls are shown in the upper right corner.
